# Pre-Treatment of Transplant Donors with Hydrogen Sulfide to Protect against Warm and Cold Ischemia-Reperfusion Injury in Kidney and Other Transplantable Solid Organs

**DOI:** 10.3390/ijms24043518

**Published:** 2023-02-09

**Authors:** Liam McFarlane, Pierce Nelson, George J. Dugbartey, Alp Sener

**Affiliations:** 1 Matthew Mailing Center for Translational Transplant Studies, London Health Sciences Center, Western University, London, ON N6A 5A5, Canada; 2 Department of Microbiology & Immunology, Schulich School of Medicine & Dentistry, University of Western Ontario, London, ON N6A 5C1, Canada; 3 Department of Surgery, Division of Urology, London Health Sciences Center, Western University, London, ON N6A 5A5, Canada; 4 Multi-Organ Transplant Program, London Health Sciences Center, University Hospital, London, ON N6A 5A5, Canada; 5 Department of Pharmacology and Toxicology, School of Pharmacy, College of Health Sciences, University of Ghana, Legon, Accra P.O. Box LG43, Ghana

**Keywords:** ischemia-reperfusion injury (IRI), solid organ transplantation (SOT), pre-treatment, hydrogen sulfide (H_2_S), sodium thiosulfate (STS)

## Abstract

Ischemia-reperfusion injury (IRI), a pathological condition resulting from prolonged cessation and subsequent restoration of blood flow to a tissue, is an inevitable consequence of solid organ transplantation. Current organ preservation strategies, such as static cold storage (SCS), are aimed at reducing IRI. However, prolonged SCS exacerbates IRI. Recent research has examined pre-treatment approaches to more effectively attenuate IRI. Hydrogen sulfide (H_2_S), the third established member of a family of gaseous signaling molecules, has been shown to target the pathophysiology of IRI and thus appears to be a viable candidate that can overcome the transplant surgeon’s enemy. This review discusses pre-treatment of renal grafts and other transplantable organs with H_2_S to mitigate transplantation-induced IRI in animal models of transplantation. In addition, ethical principles of pre-treatment and potential applications of H_2_S pre-treatment in the prevention of other IRI-associated conditions are discussed.

## 1. Introduction

Solid organ transplantation (SOT) is the preferred therapy for end-stage organ disease. Despite its benefits, the long-term success of SOT is hampered by the incidence of ischemia-reperfusion injury (IRI). IRI is an inevitable pathological condition resulting from temporary cessation of blood flow to a tissue (warm ischemia) during graft procurement, followed by hypothermic preservation (cold ischemia) and restoration of blood flow (reperfusion) during transplantation of the graft [1]. Current mitigation strategies against IRI include static cold storage (SCS) of organ grafts at 4 °C in clinically standard preservation solutions, such as the University of Wisconsin (UW) solution [2]. While SCS is effective in reducing the metabolic demand of the graft and thereby minimizing the extent of IRI and keeping the graft in a usable state, development of IRI remains unavoidable [3]. The duration of ischemic cold storage correlates with delayed graft function (DGF) and reduced long-term survival of the grafts [4,5,6]. This necessitates the swift usage of organ grafts and as a result, thousands of organs are discarded each year [7]. This clinical problem suggests that better preservation techniques are needed to improve graft quality and help combat the global donor organ shortage crisis [7]. Many additional strategies to mitigate IRI in SOT have been proposed and tested in experimental models [3]. These include the pharmacological modification of organ preservation solutions, ischemic preconditioning (brief periods of ischemia to protect against subsequent long ischemic periods), and mechanical perfusion [8,9,10,11]. In particular, the strategy of pre-treating donor organs before procurement has gained credit as a prospective solution. A study by Niemann et al. [12] enrolled 394 kidney donors after neurological death to investigate the role of mild hypothermic (34–35 °C) pre-treatment on renal transplant outcomes. Body temperature of neurologically deceased donors was either actively maintained at 37 °C as a control or cooled to 34 °C, with these target temperatures being maintained until organ procurement. Interestingly, the rate of DGF was significantly lower in transplanted kidneys from the hypothermic group. Another trial investigating the potential of pre-treatment is currently underway in Canada, where neurologically deceased kidney donors are administered tacrolimus, a calcineurin inhibitor, intravenously 4–8 h before organ procurement [13]. The goal of this pilot trial is to determine the effectiveness of tacrolimus pre-treatment on reducing DGF through protection against IRI. Pre-treatment with other pharmacological agents, such as hydrogen sulfide (H_2_S) are currently being investigated with the goal of limiting IRI and reducing the occurrence of DGF. The clinical potential of H_2_S pre-treatment in SOT is considerable. Pre-treatment could be easily paired with either of the two most widely used mitigation strategies, SCS and machine perfusion. Extending beyond pre-treatment, the inclusion of H_2_S in the SCS preservation solution and mechanically perfused solution has been shown by our research group to benefit these respective strategies in a renal transplantation model [14,15]. In this review, we discuss the concept of treating donor animals with H_2_S before their organs are harvested for transplantation into recipient animals.

## 2. Cellular Mechanisms Underlying Ischemia-Reperfusion Injury in SOT

**Figure 1:**
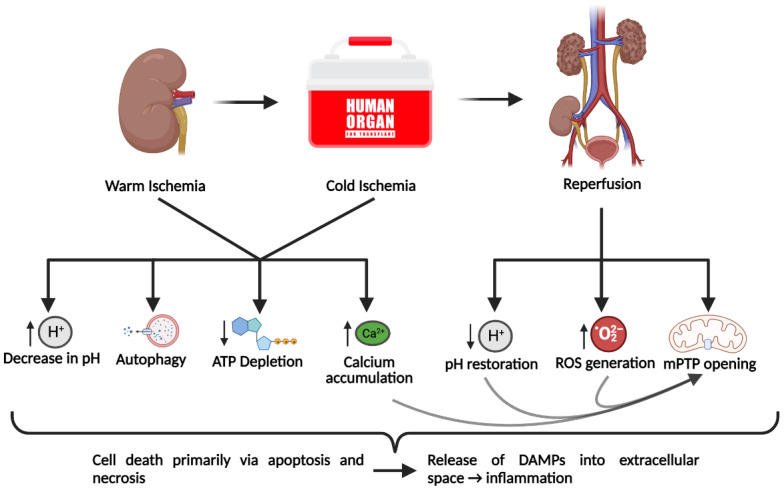
Cellular mechanisms of ischemia-reperfusion injury incurred through solid organ transplantation. The warm ischemia period begins with the interruption of perfusion to the donor organ and continues until the organ is flushed with hypothermic preservation solution, which marks the beginning of cold ischemia. The cold ischemic period typically consists of 4 °C cold storage of the procured organ and continues until the graft is implanted into the recipient. Together, these two ischemic periods lead to the generation of a pathological state that is included in the depletion of ATP due to the unavailability of oxygen, calcium accumulation, and decrease in cellular pH due to altered ion channel activity, and autophagy, which likely occurs to provide a source of energy. Subsequent reperfusion of the transplanted organ induces a paradoxical response whereby the injury is exacerbated. The restoration of blood flow rapidly restores pH levels and leads to the massive generation of reactive oxygen species (ROS), which together with the high intracellular calcium concentration can induce the opening of mitochondrial permeability transition pores (mPTP). Collectively, these effects can induce cell death, primarily via apoptosis and necrosis. Necrotic cell death releases danger-associated molecular patterns (DAMPs) into the extracellular space and leads to an inflammatory response. Figure created with BioRender.com.

IRI can occur in various organs and tissues, commonly resulting from medical procedures, including SOT. In general, IRI is characterized by oxidative stress, inflammation, mitochondrial dysfunction, and cell death primarily via necrosis or apoptosis [16,17]. However, there are certain tissue-specific characteristics of IRI that usually reflect the function of the organ in which IRI occurs [17]. The lack of perfusion to a tissue generates a hypoxic environment in the tissue. The reduction of available oxygen encourages anaerobic respiration, leading to a dramatic decrease in ATP production accompanied by a decrease in cell pH [18] (Figure 1). The high intracellular proton concentration promotes the activity of the Na^+^/H^+^ exchanger, which along with the arrest of the Na^+^/K^+^ ATPase, generates an elevated intracellular Na^+^ concentration [8]. The accumulation of cytosolic Ca^2+^ ensues due to the arrest of the ATP-dependent calcium export and the reduction in the activity of the Na^+^/Ca^2+^ exchanger [8]. As a result, mitochondrial Ca^2+^ transport is increased, resulting in elevated mitochondrial Ca^2+^ concentration [19]. Mitochondria play a critical role in the pathogenesis of IRI. During prolonged ischemic periods, protein complexes of the electron transport chain (ETC) are impaired, leading to decreased ATP production and increased electron leak [20,21]. Electron leak is responsible for the production of superoxide (O_2_^−^), which is normally eliminated through oxidation by mitochondrial superoxide dismutase (MnSOD) to H_2_O_2_ and subsequent oxidation to H_2_O by glutathione peroxidases, catalase, or peroxiredoxins [22]. This basal level of reactive oxygen species (ROS) generation is exacerbated during reperfusion, the effector phase of ischemic injury, where rapid oxidation of accumulated succinate by reverse action of the ETC drives massive ROS generation [23]. The overproduction of ROS overwhelms antioxidant enzyme activity and further contributes to ROS accumulation [24]. The resultant elevated ROS and increased mitochondrial Ca^2+^ concentration lead to the opening of the mitochondrial permeability transition pores (mPTPs) [24]. Opening of the mPTPs causes the uncoupling of oxidative phosphorylation and the release of pro-apoptotic factors into the cytoplasm and nucleus, leading to apoptosis [19]. Increased levels of inorganic phosphate, depletion of adenosine nucleotides, and rapid pH restoration upon reperfusion also contribute to mPTP opening and further cell death by apoptosis [25]. In addition to apoptosis, cell death in IRI occurs through autophagy and various forms of regulated necrosis, such as necroptosis and ferroptosis [26,27,28]. Necroptosis in particular is a major contributor to cell death in IRI, as evidenced by the protective effects of necrostatin-1 (inhibitor of necroptosis) against renal IRI [26]. Necroptosis and a more recently discovered form of regulated necrosis called ferroptosis appear to be the most prominent causes of necrotic cell death in IRI [27,29]. Cell death via necrosis induces the release of danger-associated molecular patterns (DAMPs) into the extracellular space that are normally sequestered inside the cell [30]. DAMPs, as well as ROS, induce the production of pro-inflammatory cytokines, chemokines, and the expression of endothelial adhesion molecules [18,31]. This inflammatory response causes the accumulation of neutrophils, macrophages, CD4^+^ T cells, and other immune cells in the affected tissue [18,19]. Neutrophils exacerbate cell death via the deposition of pore-forming proteins onto affected cells. Additionally, IRI induces complement activation, which further contributes to cell death and inflammation [32]. Autophagy is another form of cell death that has a unique role in the pathogenesis of IRI. It has been shown that autophagy confers protection to cells during ischemia, perhaps by providing the cell energy in the form of degraded cellular components [28]. In prolonged ischemia, however, catabolism exceeds anabolism and thus autophagy contributes to cell death [28]. Collectively, IRI induces significant cell death, ROS formation, and inflammation.

## 3. H_2_S and Its Endogenous Production

H_2_S is an established member of a family of small endogenously produced gaseous signaling molecules referred to as gasotransmitters [15,33]. This family of gaseous signaling molecules, which also includes carbon monoxide (CO) and nitric oxide (NO), plays an important role in cellular homeostasis and are being experimentally investigated in the context of organ transplantation [15,33]. H_2_S is endogenously produced via three enzymes: cystathionine beta-synthase (CBS) [34,35], cystathionine γ-lyase (CSE) [36], and 3-mercaptopyruvate sulfurtransferase (3MST) [37,38]. Both CBS and CSE are cytosolic enzymes and produce H_2_S using the amino acid L-cysteine as their substrate [34,35,36], whereas 3MST, a mitochondrial enzyme, uses 3-mercaptopyruvate (3MP) as a substrate [37,38]. In turn, 3MP may be generated via cysteine aminotransferase with α-ketoglutarate and cysteine used as substrates [38] or via D-amino acid oxidase (DAO) with D-cysteine used as a substrate [37].

Historically, H_2_S was recognized as both a human and environmental toxin [39]. At high concentrations, it inhibits complex IV of the mitochondrial ETC, which suppresses cellular proliferation and metabolism while inducing apoptosis [40,41]. However, research on H_2_S over the past several years has shown that H_2_S is an important gaseous signaling molecule with biological usefulness and therapeutic potential [39,42]. In addition to direct inhalation of the gas, H_2_S donors have been recognized as compounds that can release gaseous H_2_S in response to specific stimuli [33]. Exogenous administration of several H_2_S donors has shown great therapeutic promise in the context of IRI, such as sodium sulfide (Na_2_S), sodium hydrosulfide (NaHS), GYY4137, AP39, and sodium thiosulfate (STS) [15,43,44,45,46,47,48,49,50,51,52]. Our research group recently reviewed the use of H_2_S donors in models of transplantation-induced cold IRI [1]. These studies have largely focused on treating organs with H_2_S donors during ischemia (ischemic treatment) or after reperfusion (post-treatment). Ischemic treatment and post-treatment approaches have been shown to protect against IRI involving the brain, heart, intestine, kidneys, lungs, and pancreas [15,43,44,45,46,47,48,49,50,51,52]. The mechanisms of this protection include suppressing oxidative stress, cell death, pro-inflammatory responses, or a combination of these processes (Table 1) [15,43,44,45,46,47,48,49,50,51,52]. However, a more limited body of research has investigated the effect of pre-treating organs with H_2_S prior to ischemia (pre-treatment).

## 4. H_2_S Pre-Treatment against Cold IRI

**Figure 2:**
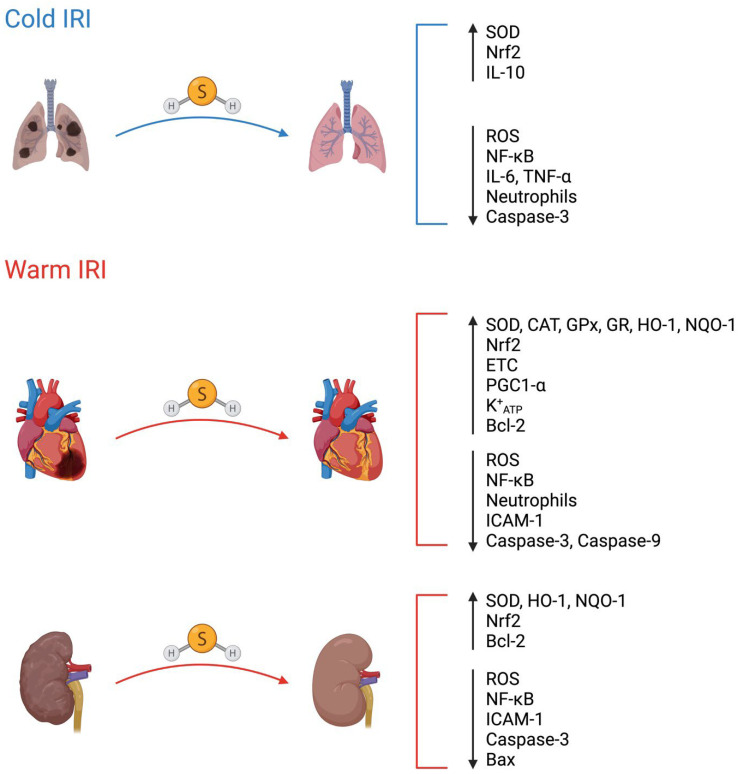
Molecular targets of H_2_S pre-treatment in cold and warm IRI. Hydrogen sulfide (H_2_S) pre-treatment regimens may protect against IRI by modifying different molecular targets. SOD: superoxide dismutase; Nrf2: nuclear factor erythroid 2-related factor 2; IL-10: interleukin-10; ROS: reactive oxygen species; NF-κB: nuclear factor kappa-light-chain-enhancer of activated B cells; IL-6: interleukin-6; TNF-α: tumor necrosis factor-alpha; CAT: catalase; GPx: glutathione peroxidase; GR: glutathione reductase; HO-1: heme oxygenase-1; NQO-1: NADPH quinone oxidoreductase-1; ETC: electron transport chain; PGC1-α: peroxisome proliferator-activated receptor gamma coactivator 1-alpha; K^+^_ATP_: adenosine triphosphate-sensitive potassium channel; Bcl2: B-cell lymphoma-2; ICAM-1: intercellular adhesion molecule-1; Bax: Bcl-2 associated X-protein. H_2_S is represented as a ball-and-stick model (sulfur in yellow; hydrogen in white; covalent bonds in grey). Figure created with BioRender.com.

Currently, there is a very limited body of research focused on the use of exogenous H_2_S pre-treatment in the context of cold IRI induced by SOT. To our knowledge, only two studies have examined the effect of exogenous H_2_S pre-treatment in models of cold IRI. Both studies have focused on lung transplantation (Figure 2).

### 4.1. H_2_S Pre-Treatment against Cold IRI in Lung Transplantation

In one study, male New Zealand white rabbits either inhaled room air supplemented with gaseous H_2_S or room air alone for two hours prior to their lungs and heart being harvested for en bloc heart-lung transplantation [53]. The heart-lung grafts were stored in preservation solution at 4 °C for 18 h, after which they were ventilated and perfused with blood from donor rabbits for two hours. The researchers found that during reperfusion, heart-lung blocs from rabbits pre-treated with H_2_S exhibited better pulmonary function as evidenced by improved oxygenation and ventilation. While ROS levels did not differ in lung biopsies taken from either the H_2_S pre-treated or control groups before SCS of the heart-lung blocs in preservation solution, ROS levels were significantly lower in lung biopsies taken during reperfusion in the H_2_S pre-treated group. This antioxidant effect of H_2_S during reperfusion is particularly important since most ROS production occurs during the reperfusion phase of IRI due to the reverse action of complex I of the ETC [23]. Interestingly, complex IV activity was significantly higher in biopsies taken from lungs in the H_2_S pre-treatment group after SCS but before reperfusion [53]. This finding may seem contradictory to the known inhibitory effect of H_2_S on complex IV of the ETC [40]. However, since H_2_S can also act as an electron donor to the ETC [54], H_2_S may have supported complex IV activity by donating electrons to the ETC to maintain ATP production during ischemia [53]. Another possible explanation is that apoptosis may have been higher in heart-lung blocs from the control group, which would have lowered complex IV activity, since apoptosis leads to the release of complex IV [53,55].

In a more recent study by Meng et al. [56] involving donation after circulatory death (DCD) lung transplantation in male Sprague Dawley rats, the researchers either deflated the lungs or inflated the lungs with air containing gaseous H_2_S or air alone for two hours. During this period of warm ischemia, lungs inflated with H_2_S had a lower metabolic rate relative to the control lungs, which aligns with a previous study that showed H_2_S can induce a hypometabolic state in mice [41]. The left lungs were then harvested and stored in preservation solution at 4 °C for 3 h followed by syngeneic transplantation into recipient rats, where reperfusion occurred for 3 h [56]. Interestingly, lungs that were inflated with H_2_S showed enhanced pulmonary function along with reduced apoptosis, inflammation, and oxidative stress. To understand the anti-inflammatory and antioxidant effects of H_2_S pre-treatment, the researchers examined the translocation of both nuclear factor kappa-light-chain-enhancer of activated B cells (NF-κB) and nuclear factor erythroid 2-related factor 2 (Nrf2) from the cytoplasm to the nucleus. NF-κB is a well-recognized transcription factor that is normally inhibited in the cytoplasm, but upon activation is translocated to the nucleus where it can upregulate the expression of pro-inflammatory genes [57]. Nrf2 is a transcription factor normally degraded in the cytoplasm but is stabilized under oxidative stress, which enables Nrf2 to translocate to the nucleus and upregulate the expression of genes involved in cellular protection [58]. As would be expected, NF-κB nuclear translocation was lower and Nrf2 nuclear translocation was higher in biopsies obtained from lungs that were inflated with gaseous H_2_S [56]. In summary, these two studies demonstrate that H_2_S pre-treatment of lung donors protects transplanted lung grafts from IRI through the suppression of apoptosis, inflammation, and oxidative stress, likely via modulation of NF-κB and Nrf2 nuclear localization.

### 4.2. H_2_S Pre-Treatment against Cold IRI in Kidney Transplantation

Although no studies have examined the impact of H_2_S pre-treatment in the context of cold IRI in kidney transplantation, a previous study showed that endogenous H_2_S production in kidney donors is associated with improved kidney function in recipients after kidney transplantation [59]. Specifically, the expression of CSE at the time of kidney graft procurement was positively associated with the glomerular filtration rate 14 days following transplantation in humans. Although this is not an example of exogenous H_2_S donor pre-treatment, this study suggests that higher levels of H_2_S in kidney transplant donors prior to graft procurement may protect kidney grafts against IRI, leading to enhanced kidney function in recipients after transplantation.

## 5. H_2_S Pre-Treatment against Warm IRI

Much of the research concerned with the effect of H_2_S pre-treatment on IRI to date has been carried out in the context of warm IRI. Although cold IRI is of considerably longer duration, warm IRI remains an inevitable consequence of SOT (Figure 1). In addition to SOT, warm IRI has implications in many other surgical procedures and pathological conditions. While pre-treatment using an agent effectively protective against warm IRI would be a tremendous asset in preventing surgically induced IRI, the unpredictable onset of ischemia in pathological conditions makes pre-treatment a less practical option. In the following section, the literature concerning the effects of H_2_S pre-treatment against warm IRI in transplantable organs will be discussed.

### 5.1. H_2_S Pre-Treatment against Warm Renal IRI

Renal IRI is a major cause of acute kidney injury (AKI) [60]. A study by Bos et al. [61] showed that inhalation of H_2_S gas prior to and during the induction of renal ischemia (which the authors referred to as pretreatment) protected mouse kidneys. In their observations, renal protection following pre-treatment with H_2_S was characterized by reduced apoptosis, inflammation, and histopathological changes. This renal protection resulted in improved renal function (as measured by serum creatinine) compared to mice that received gaseous H_2_S beginning immediately before reperfusion and the control group [61]. This protective effect of H_2_S can be attributed to the induction of hypometabolism [61] as well as the suppression of oxidative stress, reduced intracellular adhesion molecule 1 (ICAM-1) expression, and increased Nrf2 nuclear translocation [62,63,64]. The protective effects of H_2_S against warm renal IRI observed in this study cannot be attributed to pretreatment alone due to the continuation of H_2_S inhalation into the ischemic period. Still, these findings provide insight into the prospective usefulness of H_2_S pretreatment as a novel strategy to mitigate the induction of AKI in clinical settings. Utilizing H_2_S as a protective therapeutic would prove especially valuable in major surgical procedures (such as cardiac surgery), contrast dye administration, and patients with sepsis, all of which are major causes of AKI [65,66]. It could also be extended to experimental and clinical kidney transplantation in which kidney donors could be treated with H_2_S donors prior to renal graft procurement, during which a brief period of warm ischemia occurs. A summary of studies involving pre-treatment with H_2_S in cold and warm IRI is provided below in Table 2.

### 5.2. H_2_S Pre-Treatment against Warm Myocardial IRI

Warm myocardial IRI is an inevitable consequence of the restoration of blood flow following myocardial infarction (MI) and contributes to an estimated 50% of the resultant infarct size [25,67]. A significant body of research has assessed the cardioprotective potential of H_2_S pre-treatment against warm myocardial IRI. Sivarajah et al. [68] pre-treated rats with the H_2_S donor NaHS 15 min prior to left anterior descending coronary artery occlusion and subsequent reperfusion. Compared to the control, they observed a significant reduction in infarct size and attenuation of apoptosis, caspase 9 activity, NF-kB nuclear translocation, oxidative stress, myeloperoxidase activity, and neutrophil infiltration in the cardiomyocytes of the NaHS-treated group. In a similar study, pre-treatment with NaHS resulted in a more significant reduction in infarct size than NaHS post-treatment [69]. H_2_S pre-treatment could also be especially valuable in diabetes mellitus patients, who are at increased risk for myocardial ischemia and its associated mortality [70]. In *db/db* mice, the cardioprotective effect of H_2_S pre-treatment against myocardial IRI has been described [71]. Taken together, the cardioprotective effects of H_2_S pre-treatment in these experimental models demonstrate the need for further research on the potential of clinically viable H_2_S donor molecules in attenuating the damage induced by myocardial IRI. Since warm IRI also occurs during SOT, further research could provide a clinical rationale for pre-treating heart donors with H_2_S prior to heart procurement.

### 5.3. H_2_S Pre-Treatment against Warm Hepatic IRI

Ischemia-reperfusion injury is the most common cause of liver dysfunction following liver surgery [72]. Zhang et al. [73] demonstrated that the administration of NaHS to rats 5 min before the induction of hepatic ischemia reduced levels of necrotic, mitochondrial-related, and apoptotic cell death. The authors attributed this protective effect to the inhibition of mPTP opening and activation of Akt-GSK-3β signaling in hepatocytes. A similar study by Cheng et al. [74] also found NaHS to have a protective effect against hepatic IRI. In this study, NaHS was shown to reduce the expression of the pro-inflammatory cytokines TNF-α and IL-6 in addition to diminishing apoptotic cell death via the inhibition of JNK1 signaling. The protective effects of H_2_S against hepatic IRI in these models demonstrate the potential clinical utility of H_2_S administration preceding surgical procedures known to induce warm IRI in the liver, such as liver transplantation.

### 5.4. H_2_S Pre-Treatment against Warm Intestinal IRI

Intestinal IRI can result from necrotizing enterocolitis, midgut volvulus, intussusception, adhesive intestinal obstruction, sepsis, and hemodynamic shock [75]. Surgical induction of intestinal IRI is also seen as a consequence of cardiac surgery and liver or intestinal transplantation [76]. Protection against intestinal IRI was observed after H_2_S pre-treatment in a study by Liu et al. [77]. This protective effect was primarily attributed to the attenuation of mitochondrial damage induced by IRI. Additionally, the authors noted an anti-inflammatory effect, in which H_2_S hindered leukocyte rolling and adhesion in postischemic intestine. The protective effect of H_2_S against intestinal IRI observed in this study demonstrates the possible utility of H_2_S pre-treatment in instances of predictable induction of intestinal IRI, such as intestinal transplantation.

### 5.5. H_2_S Pre-Treatment against Warm Pulmonary IRI

Pulmonary IRI is a complication of surgical procedures, such as lung transplantation and cardiopulmonary bypass surgery, as well as pulmonary embolism [78]. The effectiveness of H_2_S pre-treatment against pulmonary IRI was examined in a study by Jiang et al. [79]. The authors demonstrated that the administration of GYY4137 prior to the occlusion the lung hilum in diabetic rats attenuated warm pulmonary IRI by reducing apoptosis and inflammation. These effects were attributed to the activation of the SIRT-1 pathway, which promoted Nrf2/HO-1 and eNOS-mediated antioxidant signaling pathways. If hydrogen sulfide pre-treatment does indeed provide clinically significant protection against warm pulmonary IRI, the utility of this therapeutic would benefit lung transplant and cardiopulmonary bypass surgeries tremendously.

## 6. Sodium Thiosulfate: A Clinically Viable H_2_S Donor Drug against IRI

When considering the clinical relevance of H_2_S pre-treatment against IRI, the non-viability of gaseous H_2_S demonstrates the need for clinically viable donor molecules that can exhibit therapeutic potential against IRI. While the H_2_S donor molecules used in the studies above (GYY4137, AP39, and NaHS) demonstrated protective effects in animal models of IRI, clinical translation of these compounds is years away. Alternatively, STS is an H_2_S donor molecule that could expedite clinical translation as it is already used clinically for the treatment of acute cyanide poisoning, cisplatin toxicity in cancer patients, and calciphylaxis in patients with end-stage renal disease [83,84,85,86]. Thiosulfate is produced endogenously from H_2_S through the mitochondrial sulfide oxidation pathway, which we recently reviewed [87]. Importantly, the reverse reaction also occurs whereby H_2_S is generated from thiosulfate [87]. A small body of literature has examined the effects of STS in IRI. Marutani et al. [43] demonstrated that following warm cerebral IRI, a single dose or week-long regimen of intraperitoneal STS injections improved the survival rate and neurological function in mice. In addition, Sen et al. [88] found that STS provided cardioprotective effects when administered after generating an atrioventricular fistula. These effects were accompanied by an increase in endogenous H_2_S, which was suggested to be responsible for the therapeutic effects. Recently, a clinical trial in the Netherlands examined the effects of administering STS to patients presenting with ST-segment elevation myocardial infarction prior to restoring perfusion through percutaneous coronary intervention [89]. Unfortunately, the researchers observed no effect of the STS treatment on infarct size, leading to the discontinuation of the trial [90]. However, Ravindran et al. [80] demonstrated that STS pre-treatment is protective against cell death, ROS accumulation, and mitochondrial dysfunction induced by in vitro hypoxia and re-oxygenation in cardiomyocytes. By occluding the left anterior descending artery, these researchers also observed similar protective effects of STS pre-treatment in an ex vivo model of warm IRI in rat hearts. The authors further reported that STS pre-treatment preserved mitochondrial integrity, leading to protection against warm myocardial IRI [81]. However, such protection conferred by STS was abolished in the presence of PI3K/mTOR/K_ATP_ inhibitors [82], suggesting that the mechanism underlying STS protection against myocardial IRI is at least partially via the PI3K/mTOR/K_ATP_ pathway.

## 7. Ethics and Regulations

The concept of treating an organ donor prior to graft procurement may raise several ethical issues. In the context of H_2_S donor pre-treatment, a major ethical issue would be the possible toxicity of H_2_S [39,40]. Such an ethical issue would be particularly problematic in the context of living donors, where the administration of H_2_S donor molecules could cause serious side effects in the organ donor. It must be emphasized that treatment of living donors with any class of drugs for the purpose of ameliorating transplantation outcomes in the recipient must not harm the donor in any way. Accordingly, extensive pre-clinical and clinical studies would need to be conducted to identify a safe and efficacious dose of a clinically viable H_2_S donor for administration in humans. The optimal dosage would depend on the specific H_2_S donor used since different H_2_S donors have different potencies [52]. A potential approach to facilitate the translation of H_2_S donor pre-treatment from bench to bedside would be to focus on H_2_S donors that are already approved by national health regulatory agencies, such as STS [91,92]. However, it is important to note that STS treatment is associated with adverse side effects, such as hypotension, headaches, nausea, and vomiting [91]. To confer protection against IRI, the required dose of STS may be higher than the recommended dose [91], which could lead to worsened side effects in the organ donor. Nonetheless, research focused on protection against IRI should prioritize the use of H_2_S donors clinically approved for other diseases, since such H_2_S donor drugs would be more likely to receive approval for use in transplantation. Another important factor to consider is that treating a living organ donor with a drug before procurement of the organ graft may dissuade the donor from donating the organ. The decision to donate an organ and the pre-transplantation phase can cause great psychological stress in a prospective donor [93,94,95,96]. Compounding the stress of donating an organ with receiving treatment that would likely not benefit the donor but could cause harm may discourage an organ donor from deciding to donate. For example, Pillay et al. [94] reported that stem cell donors experienced anxiety toward the possible adverse effects of being pre-treated with granulocyte-stimulating factor, which is administered to bone marrow donors before and during transplantation [97]. If H_2_S donor pre-treatment of living organ donors is to have clinical relevance, research should be conducted to assess the impact of H_2_S donor pre-treatment on an individual’s decision to donate an organ.

Shifting the focus to pre-treating deceased donors, current international approaches to consent for organ donation require donative intent, which is distinct from informed consent [98]. Donative intent is simply the requirement that an individual formally indicates their intention to donate an organ [98]. The organ donor can choose to be informed and decide to donate their organ(s) without the involvement of a healthcare team [98]. If an individual formally declares their intention to donate, then treatment of the deceased donor with a drug would not require informed consent if such an organ procurement protocol is clinically approved. However, an important consideration that must be made with respect to donation from deceased donors is the clinical relevance of pre-treating such donors. In the case of donation after brain death (DBD), such donors are declared dead based on neurologic criteria; the circulatory system in these patients remains functional [99,100]. Thus, drugs, such as H_2_S, administered to these organ donors should effectively circulate in the body. By contrast, circulatory function in DCD donors is compromised [99,100], which may impair the ability of a pre-treatment agent to act on its target organ, depending on the organ of interest. As reported in the study by Meng et al. [56], H_2_S pre-treatment of DCD donors in lung transplantation is feasible since the lungs can simply be inflated with gaseous H_2_S. However, H_2_S donor pre-treatment of DCD donors may be more challenging in the context of other organs. It is important to note that there are two major types of DCDs: uncontrolled and controlled [99,100,101]. In the context of controlled DCDs, the cessation of circulation is anticipated [99,100,101] and thus in this scenario, administration of a pre-treatment agent prior to the onset of ischemia should be possible. By contrast, pre-treatment of uncontrolled DCD donors prior to the onset of ischemia would be unattainable since there would be no prior anticipation of the circulatory cessation [99,100,101], at which point warm ischemia would take place. Despite the inability to administer treatment prior to warm ischemia, it would still be possible to administer treatment to DCD donors before the induction of cold ischemia. A major challenge would be circulating the pre-treatment agent to the organ of interest due to the lack of circulation. However, cardiovascular resuscitation (CPR) may serve as a potential option for circulating pre-treatment agents in DCD donors prior to organ harvesting and cold ischemic induction. For instance, Limkemann et al. [102] found that intravenous administration of gluconate, a cell impermeant, combined with CPR following circulatory death in rats reduced cell swelling and death along with liver injury in an ex vivo model of cold hepatic IRI. Interestingly, the authors showed that CPR distributed gluconate to various tissues in the body in a similar fashion to cardiac circulation. Although this research was conducted using gluconate as a pre-treatment agent, CPR could potentially help circulate H_2_S donor molecules in DCD donors. If any drug is to be used for pre-treating DCD donors to enhance transplantation success, the ability to circulate the drug of interest in the donor should be investigated to ensure the effectiveness of the treatment.

Collectively, pre-treating organ donors to protect against IRI and thereby enhance transplantation outcomes in recipients presents several ethical questions that must be considered. The importance of addressing these ethical and regulatory questions should be emphasized, as the concept of organ donor pre-treatment is becoming more of a clinical possibility. As previously mentioned, a clinical trial focused on pre-treating neurologically deceased kidney donors with tacrolimus to protect against renal IRI and improve post-transplantation outcomes is currently underway in Canada [13]. Importantly, this trial is funded by the Canadian Institutes of Health Research (CIHR) and has received approval from both federal and provincial health regulatory agencies [103]. The trial, referred to as the CINERGY pilot trial, may pave the way for future clinical trials focused on organ donor pre-treatment.

## 8. Conclusions

In conclusion, the alternative approach of pre-treating transplant donors with H_2_S donor compounds may enhance post-transplantation outcomes in recipients by protecting against IRI. The lack of literature surrounding H_2_S pre-treatment in the context of transplantation demonstrates a key research area with great potential. Considering that many studies have shown that H_2_S pre-treatment is protective against warm IRI, it is likely that H_2_S pre-treatment can protect against transplantation-induced cold IRI. Importantly, research in this area could enhance transplantation success and thereby improve the survival and quality of life of transplant recipients. As such, our research group is currently examining whether STS pre-treatment of donors can protect against transplantation-induced renal IRI. If a protective effect is established, the ethical implications of donor pre-treatment will need to be considered for clinical translation. Indeed, donor pre-treatment is becoming more of a clinical possibility, as evidenced by the CINERGY pilot trial, and may ultimately represent the future of transplantation.

## Figures and Tables

**Table 1:** Summary of the protective effects of exogenous H_2_S ischemic treatment and post-treatment in animal models of cold and warm IRI.

Model	H_2_S Treatment Modality	Effect of H_2_S	References
Warm cerebral IRI in mice	STS administered one minute or one minute and daily for one week after reperfusion	-Improved survival -Improved neurological function	[43]
Warm hepatic IRI in mice	Na_2_S administered during ischemia (five minutes before reperfusion)	-Reduced liver injury -Increased ratio of GSH to GSSG -Increased protein expression of Trx-1, HSP-90, and Bcl-2 -Decreased protein expression of cleaved caspase-3 -Reduced lipid peroxidation	[44]
Warm intestinal IRI in mice	NaHS administered during reperfusion	-Increased mesenteric perfusion -Reduced intestinal mucosal damage -Decreased levels of IL-6, IL-9, IL-10, VEGF, FGF-2, MIP-1α, eotaxin, IP-10, MIP-2, G-CSF, KC in intestinal tissue -Effects of H_2_S mediated through endothelial nitric oxide synthase	[45]
Warm myocardial IRI in mice	Na_2_S administered during reperfusion	-Reduced myocardial injury and infarct size -Reduced level of IL-1β and apoptosis in cardiac tissue -Increased cardiac function -Reduced leukocyte infiltration -Increased efficiency of ETC complexes I and II	[46]
Cold pancreatic IRI in pigs	AP39 administered during ischemia (preservation solution supplemented with AP39)	-Decreased ROS production -Increased mitochondrial membrane polarization -Increased ATP production -Decreased expression of IL-1β and TNF-α -Improved islet function in recipient mice following xenogeneic transplantation	[47]
Cold pulmonary IRI in rabbits (ex vivo)	NaHS administered during reperfusion	-Decreased ROS production	[48]
Cold renal IRI in rats	NaHS administered during ischemia (preservation solution supplemented with NaHS)	-Improved recipient survival and renal function -Reduced renal tissue apoptosis and necrosis -Reduced leukocyte infiltration and expression of IFN-γ and ICAM-1	[49]
Warm renal IRI in mice	NaHS administered daily beginning two days after reperfusion	-Reduced renal tubule damage -Improved renal function and recovery of recipient body weight -Increased tubular epithelial cell and decreased interstitial cell proliferation -Reduced renal fibrosis -Decreased ROS production, ratio of GSSG to GSH, and Nox4 expression -Increased MnSOD and catalase expression	[50]
Warm renal IRI in rats	AP39 administered during ischemia	-Improved renal function -Decreased ROS production -Decreased neutrophil infiltration and IL-12 levels -Decreased apoptosis	[51]
Cold renal IRI in rats	STS administered during ischemia (preservation solution supplemented with STS)	-Improved recipient survival and renal function -Decreased apoptosis and necrosis -Decreased KIM-1, IFN-γ, TNF-α, IL-6, Bax, Caspase-3, and JNK2 expression -Increased PGC-1α, NDUFB8, SDHB, ERK1, and ERK2 expression -Decreased macrophage and neutrophil infiltration	[15]
Cold renal IRI in rats	AP39 administered during ischemia (preservation solution supplemented with AP39)	-Improved recipient survival and renal function	[52]

GSH: reduced glutathione; GSSG: oxidized glutathione; Trx-1: thioredoxin-1; 90-kDa heat shock protein (HSP-90); Bcl-2: B-cell lymphoma-2; IL-6: interleukin-6; IL-9: interleukin-9; IL-10: interleukin-10; VEGF: vascular endothelial growth factor; FGF-2: fibroblast growth factor 2; MIP-1α: macrophage inflammatory protein-1 alpha; IP10: C-X-C ligand 10; MIP-2: macrophage inflammatory protein 2; G-CSF: granulocyte-colony stimulating factor; KC: C-X-C ligand 1; IL-1β: interleukin-1 beta; ETC: electron transport chain; ROS: reactive oxygen species; ATP: adenosine triphosphate; TNF-α: tumor necrosis factor-alpha; IFN-γ: interferon gamma; ICAM-1: intercellular adhesion molecule-1; Nox4: NADPH oxidase 4; MnSOD: manganese superoxide dismutase; IL-12: interleukin-12; KIM-1: kidney injury molecule-1; Bax: Bcl-2 associated X-protein; JNK2: c-Jun N-terminal kinase 2; PGC-1α: Pparg coactivator 1 alpha; NDUFB8: NADH dehydrogenase [ubiquinone] 1 beta subcomplex subunit 8; SDHB: succinate dehydrogenase [ubiquinone] iron-sulfur subunit; ERK1: mitogen-activated protein kinase 1; ERK2: mitogen-activated protein kinase 2.

**Table 2:** Summary of protective effects of exogenous H_2_S pre-treatment in animal models of cold and warm IRI.

Model	H_2_S Pre-Treatment Modality	Effect of H_2_S	References
Cold pulmonary IRI in rabbits	Inhalation of H_2_S for 2 h prior to procurement	-Better pulmonary function in recipient -Lower ROS production following reperfusion	[53]
Cold pulmonary IRI in rats	Inflation of procured lung with H_2_S for 2 h before SCS	-Reduced apoptosis, inflammation, and oxidative stress -Reduced NF-kB nuclear localization -Increased Nrf2 nuclear localization	[56]
Warm renal IRI in mice	Inhalation of H_2_S for 30 min prior to ischemia	-Reduced impairment of kidney function, apoptosis, inflammation, and degree of structural damage -Attributed protective effect to hypometabolism induced by H_2_S	[61]
Warm renal IRI in rats	NaHS administered 10 min before ischemia	-Reduced levels of plasma creatinine, blood urea nitrogen, renal malondialdehyde concentration, and increased superoxide dismutase activity	[62]
Warm renal IRI in rats	NaHS administered daily for 35 days before ischemia	-Decreased NF-kB concentration -Downregulation of ICAM-1 expression	[63]
Warm renal IRI in mice	GYY4137 administered for 2 consecutive days before ischemia	-Elevated Nrf2 nuclear translocation	[64]
Warm myocardial IRI in rats	NaHS administration 15 min prior to ischemia	-Reduced infarct size -Reduced apoptosis, caspase 9 activity, NF-kB nuclear translocation, oxidative stress, myeloperoxidase activity, and neutrophil infiltration	[68]
Warm myocardial IRI in rats	NaHS administered 1 day before ischemia	-Cardioprotection through a PKC-dependent mechanism -Pre-treatment provided a greater protective effect than post-treatment	[69]
Warm myocardial IRI in *db/db* mice	Na_2_S administered 24 h or daily injection for 7 days before ischemia	-Infarct size relative to area at risk was reduced in both treatment regiments compared to vehicle control, but was 51% more effective in 7 day treatment than acute treatment.	[71]
Warm myocardial IRI in rat heart (ex vivo)	STS administration 15 min before ischemia	-Reduced apoptosis and ROS levels. -Preserved mitochondrial function	[80]
Warm myocardial IRI in rat heart (ex vivo)	STS administration 15 min before ischemia	-Improved activity of ETC complexes I-IV -Elevated PGC1α expression	[81]
Warm myocardial IRI in rat heart (ex vivo)	STS administration 15 min before ischemia	-Protective effects abolished in the presence of PI3K/mTOR/KATP inhibitors	[82]
Warm hepatic IRI in rats	NaHS administration 5 min before ischemia	-Reduced necrosis, mitochondrial-related cell death and apoptosis -Inhibited mPTP opening and activation of Akt-GSK-3β signaling	[73]
Warm hepatic IRI in mice	NaHS administration 30 min before ischemia	-Reduced expression of TNF-α and IL-6 -Reduced apoptosis through inhibiting JNK1 signaling	[74]
Warm intestinal IRI in rats	NaHS administered 24 h before ischemia	-Prevented postischemic mitochondrial dysfunction) in a BK_Ca_ channel-dependent manner -Reduced leukocyte rolling and adhesion in postischemic intestine	[77]
Warm pulmonary IRI in rats	GYY4137 administered 1 h before ischemia	-Promoted Nrf2/HO-1 and eNOS-mediated antioxidant signaling pathways.	[79]

ROS: reactive oxygen species; NF-κB: nuclear factor kappa B; Nrf2: nuclear factor erythroid 2–related factor 2; ICAM-1: intercellular adhesion molecule 1; PKC: protein kinase C; ETC: electron transport chain; PGC1α: peroxisome proliferator-activated receptor gamma coactivator 1-alpha; PI3K: phosphoinositide 3-kinase; mTOR: mammalian target of rapamycin; KATP: ATP-sensitive potassium; mPTP: mitochondrial permeability transition pore; Akt: protein kinase B; GSK-3β: glycogen synthase kinase-3 beta; TNF-α: tumor necrosis factor-alpha; IL-6: interleukin-6; JNK1: c-Jun N-Terminal Protein Kinase 1; BKCa channel: calcium-activated, large conductance potassium channel; HO-1: heme oxygenase-1; eNOS: endothelial nitric oxide synthase.

## Data Availability

Not applicable.
